# Long noncoding RNA SATB1-AS1 contributes to the chemotherapy resistance through the microRNA-580/ 2’-5’-oligoadenylate synthetase 2 axis in acute myeloid leukemia

**DOI:** 10.1080/21655979.2021.1971508

**Published:** 2021-09-13

**Authors:** Hong Zhou, Xiaofeng Jia, Fan Yang, Pengfei Shi

**Affiliations:** a Department of Hematology, Affiliated Hangzhou First People’s Hospital, Zhejiang University School of Medicine, Hangzhou, Zhejiang, P.R. China; b College of Life Sciences, China Jiliang University, Hangzhou, Zhejiang, P.R. China

**Keywords:** Acute myeloid leukemia, long noncoding RNA SATB1-AS1, microRNA-580, OAS2, chemotherapy resistance, GSK3β/β-catenin pathway

## Abstract

Acute myeloid leukemia (AML) represents a hematopoietic cancer with an invasive property. Chemoresistance blunts the therapeutic effect of chemotherapeutics in AML. Long noncoding RNAs (lncRNAs) have been implicated in chemotherapy resistance in AML. Transcriptome sequencing in the current study was applied to clarify the differentially expressed lncRNAs between peripheral blood mononuclear cells of AML and normal samples. The expression of special AT-rich sequence binding protein 1 antisense RNA 1 (SATB1-AS1) and 2ʹ-5ʹ-oligoadenylate synthetase 2 (OAS2) in AML patients was evaluated by qRT-PCR. The relationships among SATB1-AS1, microRNA-580 (miR-580) and OAS2 were investigated by dual-luciferase reporter assay. We observed that SATB1-AS1 and OAS2 were upregulated, while miR-580 was downregulated in AML patients. SATB1-AS1 depletion suppressed proliferation, and enhanced apoptosis and sensitivity of AML cells. Additionally, SATB1-AS1 promoted the expression of OAS2 by acting as a molecular sponge of miR-580 in AML. miR-580 downregulation, OAS2 overexpression and a selective glycogen synthase kinase (GSK)-3β inhibitor AR-A014418 abolished the effects of SATB1-AS1 deletion on the chemosensitivity of AML cells. In conclusion, SATB1-AS1 knockdown promotes the sensitivity of AML cells by upregulating miR-580 and downregulating OAS2 through the GSK3β/β-catenin pathway, providing new insights into the function of SATB1-AS1 as a miRNA sponge in AML.

## Introduction

Acute myeloid leukemia (AML) is characterized by infiltration of the bone marrow, blood, and other tissues through proliferative, clonal and abnormally differentiated cells of the hematopoietic system [1]. The most commonly acknowledged regimen for patients with AML involves 7 d of cytarabine (Cyt) simultaneously with 3 d of anthracycline antibiotics, including daunorubicin, idarubicin, or adriamycin (Adr), which improve a complete response rate from 55% to 70% [2]. However, the major challenge for incurable patients is resistance to treatment, often demonstrated as relapse from remission [3]. As a consequence, discovery of new therapeutic targets and elucidation of the mechanism of the chemotherapy resistance in AML may offer novel insights for AML treatment.

Long noncoding RNAs (lncRNAs), RNA molecules greater than 200 nt, are believed to facilitate proliferation, invasion as well as metastasis of different kinds of cell systems [4]. Most importantly, a great number of lncRNAs has been associated with chemoresistance during the treatment of numerous malignancies, including AML [5]. For instance, linc00239 promoted chemoresistance against Adr and enhanced malignance of AML cells [6]. Besides, lncRNA taurine upregulated gene 1 conferred resistance to Adr in AML *in vitro* and *in vivo* through suppressing microRNA-34a [7]. Also, the anti-chemoresistant role of HOXA cluster antisense RNA 2 in AML cells to Adr has been underscored lately [8]. Here in this report, we resorted to the transcriptome profiling technique to select differentially expressed lncRNAs in peripheral blood mononuclear cells (PBMC) from AML patients, from which a new special AT-rich sequence binding protein 1 antisense RNA 1 (SATB1-AS1) was screened out. LncRNA SATB1-AS1 was elevated by 215-fold in colorectal adenocarcinoma tissues relative to control adjacent tissues [9], while its function in AML was rarely investigated. Of note, a regulatory axis through which lncRNAs could serve as a competing endogenous RNA (ceRNA) to modulate the expression pattern and biological functions of microRNAs (miRNAs), thereby repressing the expression of target genes of the miRNA to involve in cancer development has been established [10]. Interestingly, transcriptome profiling also revealed that a mRNA 2ʹ-5ʹ-oligoadenylate synthetase 2 (OAS2) was highly expressed and correlated with SATB1-AS1 expression in AML patients. Thus, TargetScan and StarBase datasets were utilized to predict the binding miRNAs of SATB1-AS1 and OAS2. miR-580 was monitored in the intersection of the predictive results. Given these findings, we hypothesized that SATB1-AS1 may sponge miR-580 with OAS2 to regulate the event of chemoresistance of AML cells to Adr and Cyt. This study aims to investigate whether SATB1-AS1b could be a possible target for overcoming chemoresistance in AML via the miR-580/OAS2 axis.

## Materials and methods

### Patient recruitment and sample acquisition

A total of 43 patients with AML who did not receive any treatment between April 2015 and September 2017 were recruited to Affiliated Hangzhou First People’s Hospital, Zhejiang University School of Medicine for blood sample or bone marrow collection. Among them, 14 were males and 29 were females with an average age of 53.5 ± 12.6 years. The patients were diagnosed using French-American-British criteria [11]. Normal controls were fresh peripheral blood samples collected from 26 healthy participants with matched age and gender. All patients and/or their guardians signed a written informed consent prior to the sample acquisition. Our study was initiated in consistence with the *Declaration of Helsinki* under the guidance of the Ethics Committee of Affiliated Hangzhou First People’s Hospital, Zhejiang University School of Medicine.

### Transcriptome profiling

LncRNA and mRNA sequencing was carried out using the Hiseq X10 platform (Sangon Biotech, Shanghai, China). Differentially expressed lncRNAs or mRNAs were screened based on fold-change, and *p* values were measured using *t*-test (Supplementary Files-Raw data). The threshold for differentially expressed lncRNAs or mRNAs was set as foldchange ≥2.0 and *p* value ≤0.05.

### RNA extraction

Trizol reagent (Invitrogen Inc., Carlsbad, CA, USA) was applied to isolate RNA from collected tissues or cells. Briefly, RNA degradation and contamination were monitored on a 1% agarose gel. According to the manufacturer’s protocol, each tissue sample was washed three times with cold phosphate-buffered saline and incubated with 1 mL TRIzol® reagent (Thermo Fisher Scientific Inc, Waltham, MA, USA) to extract RNA. RNA concentration was measured using the Qubit® RNA Assay Kit in the Qubit® 2.0 Fluorometer (Thermo Fisher Scientific, Inc.). RNA integrity was assessed using the RNA Nano 1000 Assay Kit from Bioanalyzer 2100 System (Agilent Technologies, Santa Clara, CA, USA) [12].

### Quantitative real-time polymerase chain reaction (qRT-PCR)

To detect SATB1-AS1 expression, PrimeScript RT MasterMix (TaKaRa, Tokyo, Japan) was used to reverse the isolated RNA into single-stranded cDNA. SATB1-AS1 expression was determined using the Power SYBR Green Master Mix (Thermo Fisher Scientific Inc) kit. For detection of miR-580 expression, cDNA was generated from extracted total RNA with the help of the Mir-X miRNA first-strand synthesis kit (TaKaRa), and the subsequent reverse transcription PCR was carried out with a TaqMan MicroRNA Analysis Kit (Applied Biosystems, Forster City, CA, USA). After that, we used ABI Prism 7500 Real-Time PCR system (Applied Biosystems) to detect expression of miR-580, SATB1-AS1 and OAS2 with U6 or glyceraldehyde-3-phosphate dehydrogenase (GAPDH) as the internal reference. The primers are listed in Table 1.

**Table 1.** Primers for qRT-PCR

Targets	Sequence (5ʹ–3ʹ)
STAB1-AS1	Forward: CCCGCACGATTTCATTGAAC
STAB1-AS1	Reverse: GGCGGATTGGAAATGAACTTC
OAS2	Forward: CTGACCAGCTGCACTCG
OAS2	Reverse: CTCAGGCGCTGATCTCTTG
GAPDH	Forward: TGGTTAAGCTCTTACACCCAC
GAPDH	Reverse: CACGAACAAGCAACTGAACTAG
miR-580	Forward: CACTCCTTTCATACGCTTTTCTG
miR-580	Reverse: ATGCTGGTAACACTGTGGTC
U6	Forward: GAAGGTGAAGGTCGGAGTC
U6	Reverse: GAAGATGGTGATGGGATTTC

**Note**: qRT-PCR, quantitative real-time polymerase chain reaction; SATB1-AS1, special AT-rich sequence binding protein 1 antisense RNA 1; OAS2, 2ʹ-5ʹ-oligoadenylate synthetase 2; miR-580, microRNA-580; GAPDH, glyceraldehyde-3-phosphate dehydrogenase.

### Construction of transfection plasmids

Small interfering RNA (siRNA) targeting SATB1-AS1 (si-SATB1-AS1) or its scrambled control (scramble siRNA) was designed and synthesized from Invitrogen. miR-580 inhibitor, inhibitor control (Mock), pcDNA-SATB1-AS1, pcDNA-OAS2 and pcDNA empty vector (Empty Vector) were purchased from GenePharma (Shanghai, China). Lipofectamine 2000 (Invitrogen) was used to transiently transfect the constructed oligonucleotides or plasmids into HL60 and OCI-AML5 cells. Sequence information is shown in Table 2.

**Table 2.** siRNA or inhibitor sequence used for transfection

Symbol	Sequence (5–3ʹ)
si-SATB1-AS1 guide	AAACAACAAGCAACAAGAGGA
si-SATB1-AS1 passenger	CUCUUGUUGCUUGUUGUUUGG
miR-580 inhibitor	AUUACUAAGUAGUCUGAGUCUA

**Note**: si, small interfering RNA; SATB1-AS1, special AT-rich sequence binding protein 1 antisense RNA 1; miR-580, microRNA.

### Cell culture and treatment

Human AML cell lines KG-1a, THP-1, HL60, OCI-AML3, OCI-AML5 and human bone marrow stromal cell line HS-5 were purchased from ATCC (Manassas, VA, USA). The cells were exposed to Roswell Park Memorial Institute (RPMI) 1640 medium (Gibco, Grand Island, NY, USA) containing 10% inactivated fetal bovine serum (FBS; Gibco), 100 μg/mL streptomycin and 100 U/mL penicillin (Sigma-Aldrich Chemical Company, St Louis, MO, USA) with 5% CO_2_ at 37°C. The cells used in all experiments were in the logarithmic phase of growth.

### Establishment of AML drug-resistant cell lines

HL60 cells were treated with different concentrations of Adr, and OCI-AML5 cells with different concentrations of Cyt (Sigma-Aldrich). Adr-resistant HL60 cells (defined as HL60/Adr) and Cyt-resistant OCI-AML5 cells (defined as OCI-AML5/Cyt) were established in our laboratory with parental HL60 and OCI-AML5 cells through continuous exposure to progressively increased Adr and Cyt concentrations over a period of 8 months. Subsequently, the CellTiter-Glo kit was used to identify the successful establishment of drug-resistant cell lines.

### CellTiter-Glo luminescent assays

At the 24^th^ h after drug treatment, the viability of HL60 and OCI-AML5 cells was tested following the manufacturer’s protocols of the CellTiter-Glo® kit (Promega, Madison, WI, USA) [13].

### Flow cytometry

Apoptosis was assessed with an Annexin V-fluorescein isothiocyanate (FITC) cell apoptosis detection kit (BD Biosciences, San Jose, CA, USA). In short, 200 μL binding buffer was used to resuspend the treated cells. These cells were then dual-stained for 15 min in the dark using propidium iodide (PI) and FITC. Finally, the apoptosis rate was identified by a flow cytometer (BD Biosciences) [14].

### Hoechst 33258 staining

The cells were stained according to the instructions of a Hoechst 33258 kit (Beyotime Biotechnology Co., Ltd., Shanghai, China) and observed under a fluorescence microscope (×400, Leica Microsystems GmbH, Wetzlar, Germany). The apoptotic cells in each well were counted in five different fields. The rate of apoptosis is calculated as the percentage of apoptotic cells obtained for each field divided by the total number of cells for each field[14].

### Colony formation assay

The cell suspension was seeded into a 6-well plate at about 5 × 10^2^ cells each well. Subsequently, the cells in each well were cultured with 2 mL RPMI-1640 medium, with the medium renewed every 48 h. After 10 d, the cells were fixed with formaldehyde and stained with crystal violet, and the number of colonies formed in each well was counted [15].

### 5-Ethynyl-2ʹ-deoxyuridine (EdU) assay

An EdU luminescence kit (iFluor 647, Abcam Inc., Cambridge, UK) was applied for cell proliferation measurement in strict accordance with the instructions. The stably transfected cells (5 × 10^3^ cells/well) were cultured in a 96-well petri dish for 24 h. Then, the cells in each well were cultured with 100 μL culture medium containing 20 μm EdU at 37°C for 2 h. The number of EdU positive cells was observed and calculated by a fluorescence microscopy (Olympus Optical Co., Ltd., Tokyo, Japan) [16].

### Detection of lactate dehydrogenase (LDH) activity

HL60 and OCI-AML5 parental cells and drug-resistant cells were tested for cytotoxicity using an LDH cytotoxicity assay kit (Dojindo, Kumamoto, Japan) after drug treatment. In brief, after cell lysis, intracellular LDH activity was determined by adding 1% Triton X-100, and the optical density (OD) value at 340 nm was analyzed using an automatic biochemistry analyzer. LDH release represents the ratio of LDH in the medium relative to total LDH [17].

### Tumorigenicity assay in vivo

A total of 24 athymic nude (nu/nu) mice (aged 4 weeks, weighing 22 ± 5 g) were purchased from Charles River (Wilmington, MA, USA). All experiments involving animals were authorized by the Animal Care and Use Committees of Affiliated Hangzhou First People’s Hospital, Zhejiang University School of Medicine. A total of 4 × 10^6^ HL60 or OCI-AML5 cells were subcutaneously injected into each mouse (*n* = 6 in each group, half male and half female). The tumors were allowed to grow for 10 d (about 50 mm^3^ in diameter) before any intervention was initiated. The mice were treated with Adr (5 mg/kg) [18,19] or Cyt (100 mg/kg) [20,21] by oral tube feeding on d 6 and 24. The mouse weight was recorded once every 6 d. After 30 d, the mice were euthanized by excessive injection of pentobarbital sodium at 100 mg/kg, and their tumors were harvested. The tumor size and weights were measured to calculate the mean value in each group.

### Immunohistochemistry

The immunohistochemical staining was carried out following a previous report [22] using the antibody against Ki-67 (ProteinTech Group, Chicago, IL, USA). In brief, the sections were water-bathed in a citrate buffer solution (pH = 6.0) at 100°C for 20 min for antigen retrieval. The score for immunohistochemical staining was obtained by multiplying the percentage of positive cells with the staining intensity following the method described by a previous literature [23].

### Fluorescence in situ hybridization (FISH)

We performed FISH as per instructions provided by the manufacturer (Guangzhou RiboBio Co., Ltd., Guangzhou, Guangdong, China). In brief, a SATB1-AS1-specific probe was designed, synthesized and incubated with the cells overnight at 4°C. GAPDH and U6 were applied to identify the cytoplasm and nucleus [24].

### Dual-luciferase reporter assay

The wild-type (WT) or mutant-type (MT) fragments of SATB1-AS1 or OAS2 3ʹuntranslated region (3ʹUTR) containing miR-580 target sequences were synthesized and inserted into the pGL3 promoter vector (Promega). These sequences were named SATB1-AS1-wt, SATB1-AS1-mut, OAS2 3ʹ-UTR-wt and OAS2 3ʹ-UTR-mut, respectively. The above vectors, miR-580 mimic or mimic control were co-transfected into 293T cells by using Lipofectamine 2000 (Invitrogen). The cells were collected 48 h after induction. The luminescence was assessed with a dual luciferase reporter gene detection system (Promega). The results were then normalized to Renilla luciferase activity [25].

### Immunoblotting

The total protein was extracted from the treated cells using radioimmunoprecipitation assay lysis buffer (Beyotime), separated by 10% sodium dodecyl sulfate-polyacrylamide gel electrophoresis and then electroblotted onto a polyvinylidene difluoride membrane (Millipore, Billerica, MA, USA). After the immersion of the membranes in 5% skimmed milk for 2 h at 25°C, the membranes were incubated with antibodies to OAS2 (ab195968, Abcam), β-catenin (Santa Cruz Biotechnology, Santa Cruz, CA, USA), GSK3β (ab32391; Abcam) and GAPDH (Sangon Biotech) at 4°C overnight. The blots were then incubated separately with horseradish peroxidase-coupled secondary antibody (Santa Cruz Biotechnology). The bands were measured by the enhanced chemiluminescence system (Pierce, Rockford, IL, USA) [26].

### Statistics

Statistical analyses were carried out using the SPSS 21.0 software (IBM Corp. Armonk, NY, USA). Numerical data were shown as mean ± standard deviation (SD) after Kolmogorov–Smininov test for normal distribution. Unpaired *t* test or one-way or two-way analysis of variation (ANOVA) was applied to compare the differences between two groups or among multiple groups, followed by Tukey’s multiple comparisons test. Significant differences were defined by a *p* value of <0.05.

## Results

In this study, we aimed to explore the effects of the lncRNA SATB1-AS1 on the chemosensitivity of AML cells to Adr and Cyt. We hypothesized that knockdown of SATB1-AS1 inhibited the proliferation and promoted the apoptosis of AML cells, and enhanced the chemosensitivity of AML cells via the miR-580/OAS2 axis and the GSK3β/β-catenin signaling pathway.

### SATB1-AS1 is highly expressed in AML and is associated with drug resistance

**Figure 1.**
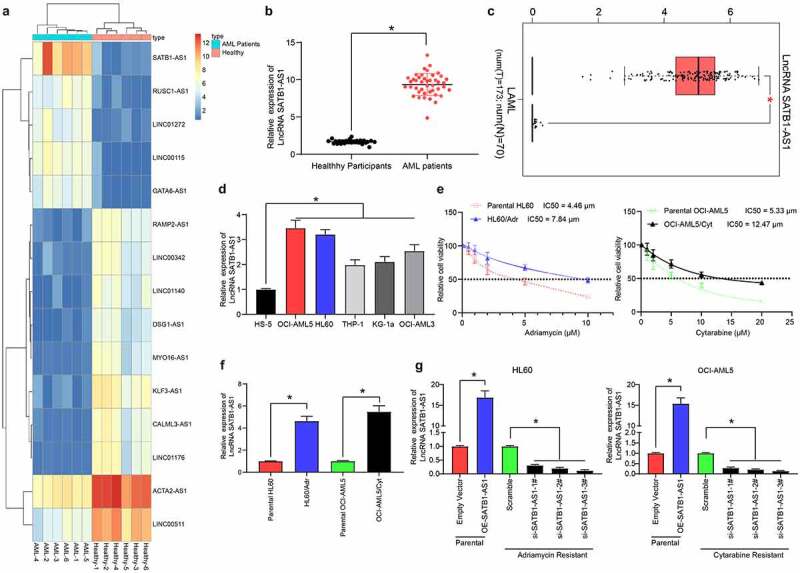
SATB1-AS1 is upregulated in PBMC of AML patients. (a) Transcriptome analysis of 15 differentially expressed lncRNAs in PBMC from six healthy subjects and six AML patients. (b) the expression of SATB1-AS1 in PBMC from 26 healthy subjects and 43 AML patients measured by qRT-PCR. (c) SATB1-AS1 expression in AML in TCGA database predicted by a bioinformatics website GEPIA (http://gepia.cancer-pku.cn/index.html). (d) SATB1-AS1 expression in human bone marrow stromal cells HS-5 and AML cell lines measured by qRT-PCR. HL60/Adr and OCI-AML5/Cyt cells were exposed to different concentrations of Adr (0, 5 and 10 μM) and Cyt (0, 5, 10, 15, 20 and 25 μM). (e) The IC50 value of the cells determined by CellTiter-Glo assay. (f) The expression of SATB1-AS1 in parental and drug-resistant cells detected by qRT-PCR. The constructed SATB1-AS1 overexpression plasmid (empty vector as control) and the siRNAs targeting SATB1-AS1 (Scramble as control) were transfected into parental cells and drug-resistant cells, respectively. (g) The expression of SATB1-AS1 in parental and drug-resistant cells at the 48th h after transfection detected by qRT-PCR. Each assessment was fulfilled in triplicate with 3-time repetition to ensure minimum deviation; statistical data were measurement data, and described as mean ± standard deviation; Unpaired *t* test (panel b) or one-way (panels d, f and g) or two-way ANOVA (panel e) was applied to compare the differences between two groups or for multiple-group comparisons, followed by Tukey’s multiple comparisons test. * *p* < 0.05

We first isolated PBMC from peripheral blood samples from AML patients and healthy controls using the Ficoll method. Subsequently, we used transcriptome analysis to identify differentially expressed lncRNAs in PMBC of AML patients as well as healthy controls. SATB1-AS1 was remarkably overexpressed in AML patients (Figure 1(a,b)), which was further validated in The Cancer Genome Atlas (TCGA) database (Figure 1(c)). Moreover, the SATB1-AS1 expression in human bone marrow stromal cells HS-5 cells and AML cells was examined, which revealed that SATB1-AS1 in AML cells was promoted relative to that in HS-5 cells (Figure 1(d)). Subsequently, a HL60/Adr cell line and an OCI-AML5/Cyt cell line was developed, whose successful construction was verified by CellTiter-Glo assay (Figure 1(e)). Still, the expression of SATB1-AS1 in drug-resistant cells was enhanced versus that in parental cells (Figure 1(f)). To determine the effect of SATB1-AS1 on the growth and chemo-resistance of HL60 and OCI-AML5 cells, we overexpressed SATB1-AS1 in parental cells and transfected three siRNAs targeting SATB1-AS1 into resistant cells. The expression of SATB1-AS1 was examined after 48 h of transfection. The transfection was successfully performed, and that siRNA-3# had the highest interference efficiency (Figure 1(g)).

### SATB1-AS1 knockdown promotes sensitivity of AML cells to Adr and Cyt

**Figure 2.**
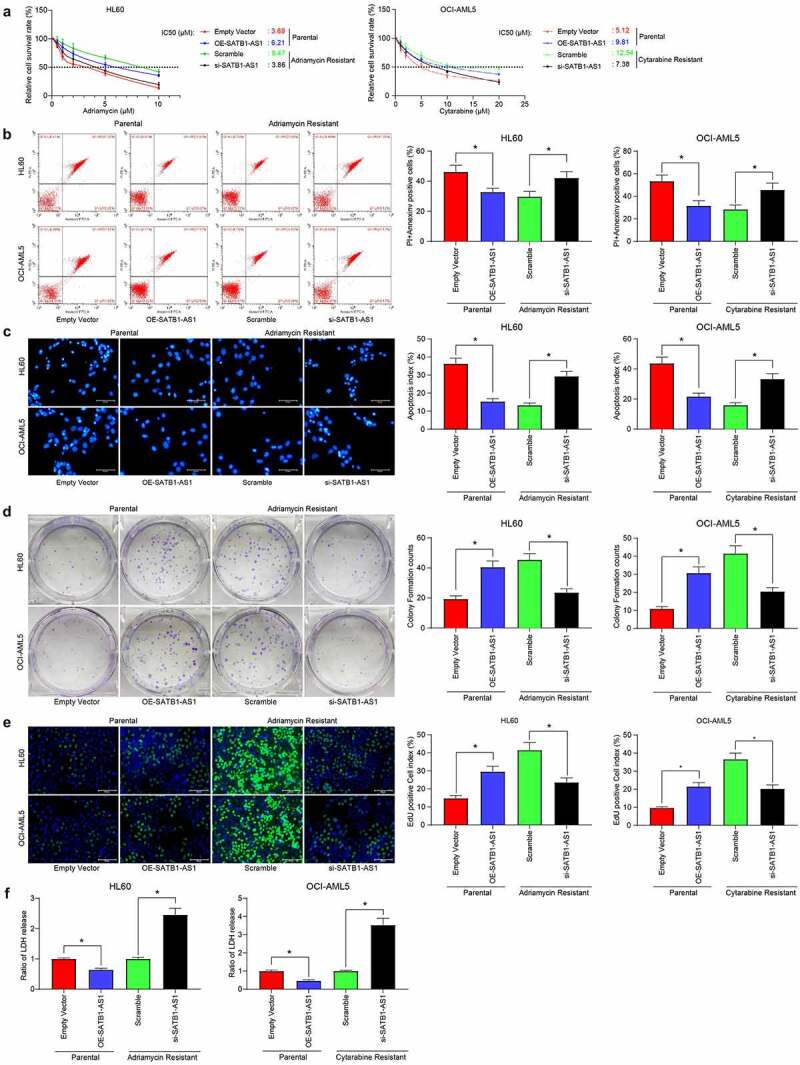
Downregulation of SATB1-AS1 mitigates the chemoresistance of AML cells *in vitro*. (a) The IC50 values of parental cells overexpressing SATB1-AS1 and of HL60/Adr and OCI-AML5/Cyt cells harboring silencing SATB1-AS1 measured by CellTiter-Glo assay. Adr (5 μM) was used to treat HL60 parental cells and resistant cells and 10 μM Cyt to treat OCI-AML5 parental cells and resistant cells, respectively. (b) Cell apoptosis tested by flow cytometry. (c) Hoechst 33,258 staining for cell apoptosis. (d) Cell proliferation evaluated by colony formation assay. (e) EdU staining for cell viability. (f) AML cell cytotoxicity evaluated by an LDH kit. Each assessment was done in triplicate with 3-time repetition to ensure minimum deviation; statistical data were measurement data, and described as mean ± standard deviation; one-way (panels b–f) or two-way ANOVA (panel a) was applied for multiple-group comparisons, followed by Tukey’s multiple comparisons test. * *p* < 0.05

We examined the changes in the median inhibition concentration (IC50) values of AML parental cells and drug-resistant cells to Adr or Cyt by CellTiter-Glo. After silencing of SATB1-AS1, the sensitivity of HL60/Adr to Adr or OCI-AML5/Cyt to Cyt was significantly increased, whereas after overexpression of SATB1-AS1 in the parental cells, the resistance of the cells to the drugs was significantly increased (Figure 2(a)). Subsequently, we used 5 μM Adr and 10 μM Cyt to treat parental and drug-resistant cells. After overexpression of SATB1-AS1, apoptotic cells induced by drug treatment in parental cells were reduced, whereas SATB1-AS1 knockdown in HL60/Adr and OCI-AML5/Cyt cells significantly promoted apoptosis induced by drug treatment (Figure 2(b,c)). We further assessed the viability changes of parental cells and drug-resistant cells after drug treatment by colony formation assays and EdU staining. After overexpression of SATB1-AS1, parental cell viability was significantly increased, whereas after reduction of SATB1-AS1 expression in drug-resistant cells, the viability was significantly inhibited (Figure 2(d,e)). Additionally, the detection of LDH activity presented that overexpression of SATB1-AS1 significantly reduced the cytotoxicity of drugs to AML parental cells, whereas inhibition of cytotoxicity significantly increased the SATB1-AS1 of drugs to resistant cells (Figure 2(f)).

**Figure 3.**
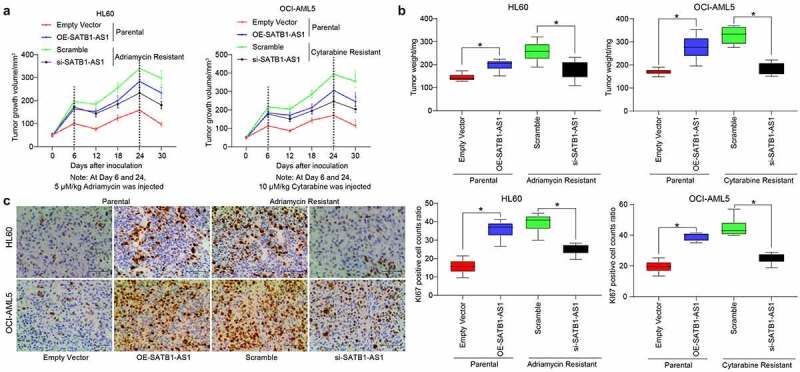
Downregulation of SATB1-AS1 mitigates the chemoresistance of AML cells *in vivo*. Each group of HL60 and OCI-AML5 parental cells and resistant cells were diluted with 2 mL normal saline (4 × 10^6^ cells) and injected under the armpit of nude mice. The tumor volume was measured every 6 d after the tumor volume grew to 50 mm^3^, and the mice were euthanized after 30 d with overdose of pentobarbital sodium (100 mg/kg). On the 6th and 24th d after the tumor volume measurement, the mice were injected with 5 mg/kg Adr and 10 mg/kg Cyt. (a) Volume of tumors in nude mouse. (b) Weight of tumors in nude mouse. (c) Immunohistochemical staining for the positive rate of KI67 in tumors. Statistical data were measurement data, and described as mean ± standard deviation (*n* = 6); one-way (panels b and c) or two-way ANOVA (panel a) was applied for multiple-group comparisons, followed by Tukey’s multiple comparisons test. * *p* < 0.05

Furthermore, we demonstrated through *in vivo* tumorigenesis experiments that si-SATB1-AS1 significantly inhibited the growth of resistant cells *in vivo* as well as drug resistance, along with diminished positive rate of KI67 in harvested tumors (Figure 3(a–c)). These experiments reflected that SATB1-AS1 knockdown contributed to the decline of the growth and resistance of HL60/Adr and OCI-AML5/Cyt cells.

### SATB1-AS1 overexpression promotes OAS2 expression

**Figure 4.**
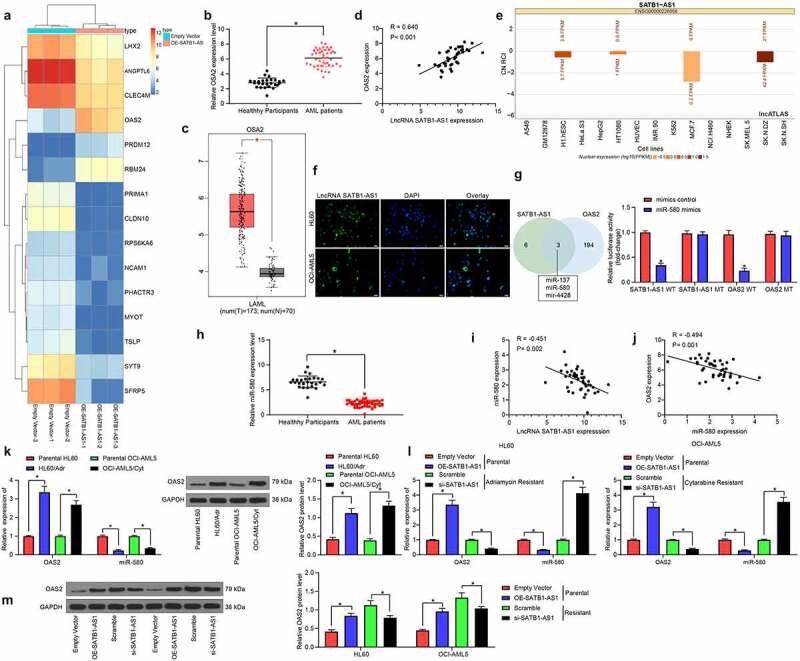
SATB1-AS1 promotes OAS2 expression by interacting with miR-580. (a) Transcriptome analysis of top 30 differentially expressed genes in HL60 cells overexpressing SATB1-AS1.; (b) The mRNA expression of OAS2 in PBMC from 26 healthy subjects and 43 AML patients measured by qRT-PCR. (c) OAS2 expression in AML in TCGA database predicted by a bioinformatics website GEPIA (http://gepia.cancer-pku.cn/index.html). (d) The correlation between SATB1-AS1 and OAS2 expression in AML patients analyzed by Pearson’s correlation test. (e) The subcellular localization of SATB1-AS1 predicted by LncAtlas (http://lncatlas.crg.eu/). (f) SATB1-AS1 subcellular localization in cytoplasm verified by FISH assay. (g) The relationship between SATB1-AS1, OAS2 and miR-580 was predicted by TargetScan and StarBase and verified by dual-luciferase reporter assays (h) The expression of miR-580 in PBMC from 26 healthy subjects and 43 AML patients measured by qRT-PCR. (i) The correlation between SATB1-AS1 and miR-580 expression in AML patients analyzed by Pearson’s correlation test. (j) The correlation between OAS2 and miR-580 expression in AML patients analyzed by Pearson’s correlation test. (k) The expression of OAS2 in HL60 and OCI-AML5 parental cells and drug-resistant cells assessed by qRT-PCR and immunoblotting. (l) The expression of OAS2 mRNA and miR-580 in HL60 and OCI-AML5 cells measured by qRT-PCR. (m) The protein expression of OAS2 in HL60 and OCI-AML5 cells measured by immunoblotting after overexpression or intervention of SATB1-AS1. Each assessment was done in triplicate with 3-time repetition to ensure minimum deviation; statistical data were measurement data, and described as mean ± standard deviation; unpaired *t* test (panels b and h) or two-way ANOVA (panels g, k–m) was applied to compare the differences between two groups or for multiple-group comparisons, followed by Tukey’s multiple comparisons test. * *p* < 0.05

With an aim to assess the role of SATB1-AS1 in AML cell resistance and growth, we performed transcriptome analysis on HL60 cells overexpressing SATB1-AS1. Some differentially expressed genes are shown in the heatmap of Figure 4(a). OAS2 was found to be upregulated in AML patients as well as in the TCGA-AML dataset (Figure 4(b,c)) and positively correlated with the SATB1-AS1 expression (Figure 4(d)). To further determine the molecular mechanism of SATB1-AS1 in AML cell resistance and growth, we predicted the subcellular localization of SATB1-AS1 in the cytoplasm (Figure 4(e)) through the LncAtlas.org website, which was substantiated by our FISH assay (Figure 4(f)). Subsequently, we resorted to TargetScan as well as StarBase to discover miR-580 as a binding miRNA for SATB1-AS1 and OAS2 (Figure 4(g)). Besides, miR-580 was significantly poorly expressed in AML patients by qRT-PCR and inversely correlated with SATB1-AS1 and OAS2 expression (Figure 4(h–j)). Consequently, we tested the OAS2 and miR-580 expression in parental cells and resistant cells and observed that the miR-580 expression in resistant cells was diminished relative to parental cells. Besides, upregulation of SATB1-AS1 repressed miR-580 expression in parental cells, while inhibition of SATB1-AS1 in the resistant cells promoted the expression of miR-580, and the expression of OAS2 was just opposite to the trend of miR-580 expression (Figure 4(k–m)). Therefore, SATB1-AS1 knockdown sensitizes AML cells to chemotherapy agents through the miR-580/OAS2 axis.

### MiR-580 inhibitor or overexpression of OAS2 reverses the effects of SATB1-AS1 knockdown on AML cell chemoresistance

**Figure 5.**
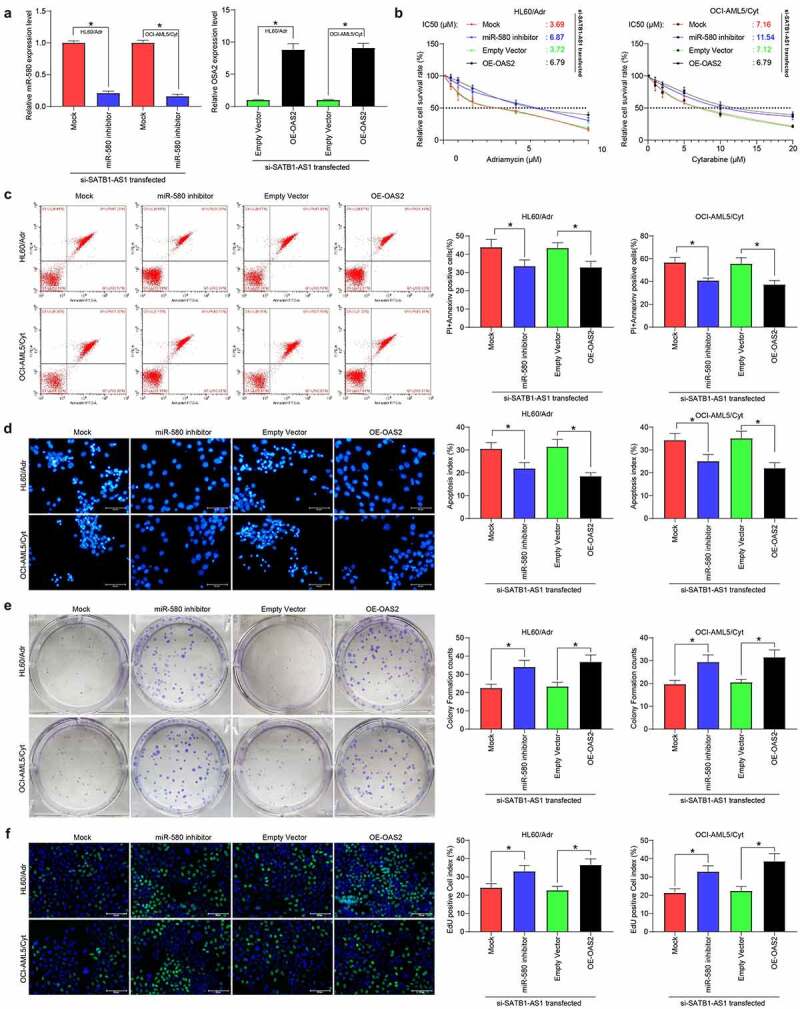
MiR-580 inhibition or OAS2 overexpression reverses the effects of SATB1-AS1 knockdown on AML cell chemoresistance. We transfected miR-580 inhibitor (Mock as a control) or OAS2 overexpression plasmid (Empty Vector as a control) in HL60/Adr cells and OCI-AML5/Cty in the presence of si-SATB1-AS1. (a) The expression of miR-580 and OAS2 in cells assayed by qRT-PCR. (b) The IC50 values of HL60/Adr and OCI-AML5/Cyt cells measured by CellTiter-Glo assay. Adr (5 μM) was used to treat HL60 parental cells and resistant cells and 10 μM Cyt to treat OCI-AML5 parental cells and resistant cells, respectively. (c) Cell apoptosis tested by flow cytometry. (d) Hoechst 33258 staining for cell apoptosis. (e) Cell proliferation determined by colony formation assay. (f) EdU staining for cell viability. Each assessment was done in triplicate with 3-time repetition to ensure minimum deviation; statistical data were measurement data, and described as mean ± standard deviation; one-way (panels a, c–f) or two-way ANOVA (panel b) was applied for multiple-group comparisons, followed by Tukey’s multiple comparisons test. * *p* < 0.05

To corroborate the ceRNA mechanism of SATB1-AS1 in AML, we transfected miR-580 inhibitor or OAS2 overexpression plasmids into HL60/Adr and OCI-AML5/Cyt cells, respectively. qRT-PCR verified the successful transfection (Figure 5(a)). We detected the change of IC50 values of AML cells to drugs by a CellTiter-Glo kit, and reducing miR-580 expression or overexpression of OAS2 significantly reduced the chemosensitivity of AML cells induced by SATB1-AS1 knockdown (Figure 5(b)). Moreover, the apoptosis rate of AML cells after drug treatments was significantly reduced, and the cell viability was significantly increased upon introduction of miR-580 inhibitor or oe-OAS2 (Figure 5(c–f)). These results suggested that miR-580 downregulation or OAS2 upregulation attenuated the effects of SATB1-AS1 knockdown on AML cell sensitivity to Adr and Cyt.

### SATB1-AS1 knockdown activates the inhibition of GSK3β on β-catenin

**Figure 6.**
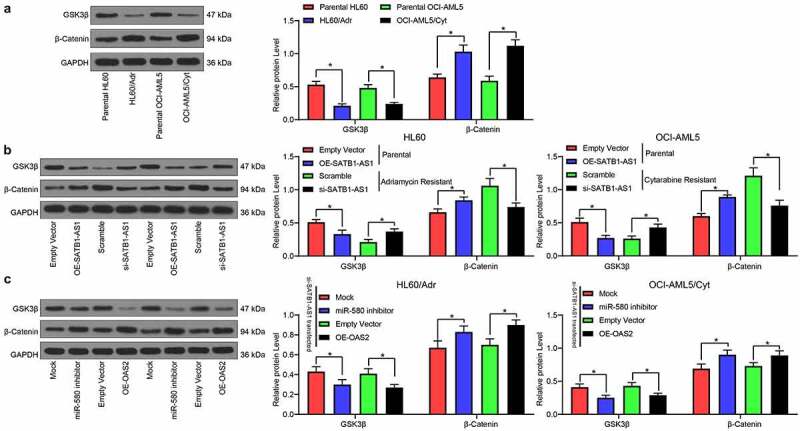
SATB1-AS1 knockdown stimulates the repressive role of GSK3β on β-catenin expression. (a) The expression of GSK3β and β-catenin in parental and drug-resistance cells detected by immunoblotting. (b) The expression of GSK3β and β-catenin in parental cells overexpressing SATB1-AS1 and drug-resistance cells with SATB1-AS1 knockdown detected by immunoblotting. (c) The expression of GSK3β and β-catenin in HL60/Adr cells and OCI-AML5/Cty cells transfected with miR-580 inhibitor or oe-OAS2 in the presence of SATB1-AS1 knockdown detected by immunoblotting. Each assessment was done in triplicate with 3-time repetition to ensure minimum deviation; statistical data were measurement data, and described as mean ± standard deviation; Two-way ANOVA was applied for multiple-group comparisons, followed by Tukey’s multiple comparisons test. * *p* < 0.05

We first examined the expression of GSK3β and β-catenin in parental cells and drug-resistant cells by immunoblotting experiments. The expression of GSK3β in drug-resistant cells was reduced, while the expression of β-catenin was enhanced (Figure 6(a)). Furthermore, after overexpression of SATB1-AS1 in parental cells, the GSK3β expression was significantly decreased, while that of β-catenin was remarkably increased (Figure 6(b)). On the contrary, silencing of SATB1-AS1 in drug-resistant cells led to opposite results. Moreover, silencing miR-580 or overexpression of OAS2 in cells harboring SATB1-AS1 knockdown significantly reduced the expression of GSK3β, whereas enhanced that of β-catenin (Figure 6(c)).

### A GSK3β inhibitor reverses the effects of SATB1-AS1 knockdown on AML cells

**Figure 7.**
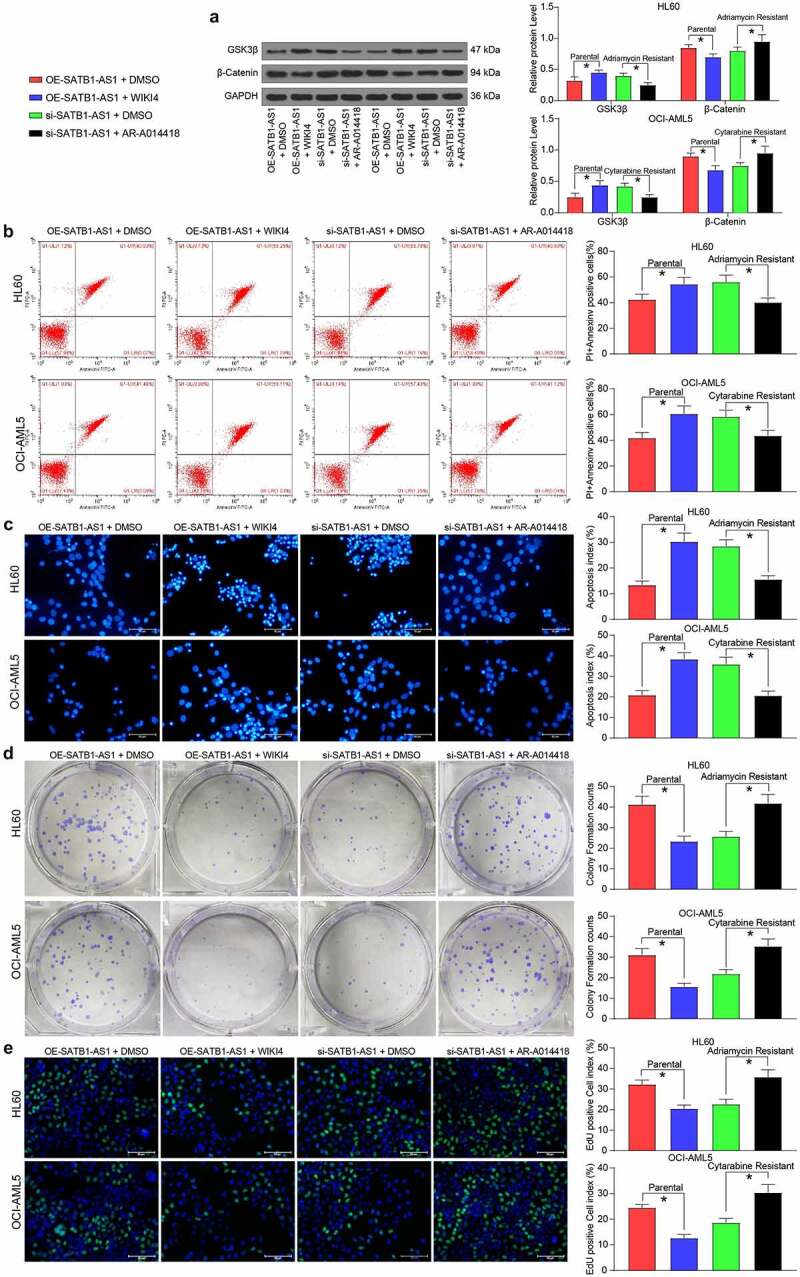
GSK3β inhibition attenuates the repressive effect of SATB1-AS1 knockdown on AML cell chemoresistance. A β-catenin specific inhibitor (WIKI4, 0.15 μM) or a GSK3β specific inhibitor (AR-A014418, 20 nM) was added to parental HL60 and OCI-AML5 cells overexpressing SATB1-AS1 or HL60/Adr cells and OCI-AML5/Cty cells with SATB1-AS1 knockdown, respectively. (a) The expression of GSK3β and β-catenin in parental and drug-resistance cells detected by immunoblotting. Adr (5 μM) was used to treat HL60 parental cells and resistant cells and 10 μM Cyt to treat OCI-AML5 parental cells and resistant cells, respectively. (b) Cell apoptosis evaluated by flow cytometry. (c) Hoechst 33258 staining for cell apoptosis. (d) Cell proliferation determined by colony formation assay. (e) EdU staining for cell viability. Each assessment was done in triplicate with 3-time repetition to ensure minimum deviation; statistical data were measurement data, and described as mean ± standard deviation; one-way (panels b–e) or two-way ANOVA (panel a) was applied for multiple-group comparisons, followed by Tukey’s multiple comparisons test. * *p* < 0.05

Next, we introduced a β-catenin specific inhibitor (WIKI4, 0.15 μM) into parental cells overexpressing SATB1-AS1, and a specific inhibitor of GSK3β (AR-A014418, 20 nM) into cells harboring SATB1-AS1 knockdown to further confirm the effect of the GSK3β/β-catenin signaling pathway on AML cell resistance. As expected, chemoresistance conferred by SATB1-AS1 in parental cells was reduced by the WIKI4 treatment, while chemosensitivity conferred by si-SATB1-AS1 in drug-resistant cells was reversed by the AR-A014418 treatment (Figure 7(a–e)).

## Discussion

Recent evidence illustrated that lncRNAs are essential for intracellular homeostasis through playing specific cellular roles, including modulation of gene transcription, development of cell cycle as well as mediation of post-transcriptional mRNA processing [27]. Even though the current combination of Cyt, a cell-phase-specific cytotoxic drug and an anthracycline remains the standard regimen in the worldwide, AML is still characterized by a dismal long-term survival with a high relapse rate resulting from chemoresistance [28]. Illuminating the molecular mechanisms of resistance to Adr and Cyt is amenable to therapeutic interventions to overcome chemoresistance in AML. Here, we monitored that a novel lncRNA, SATB1-AS1, was overexpressed in AML samples and cells. SATB1-AS1 knockdown reduced the Adr and Cyt chemoresistance and proliferation of HL60/Adr and OCI-AML5/Cyt cells, but promoted apoptosis. Besides, OAS2, a target of miR-580, was found to be enhanced by SATB1-AS1. miR-580 inhibition or OAS2 upregulation could overturn the impacts of SATB1-AS1 knockdown in HL60/Adr and OCI-AML5/Cyt cells. Therefore, SATB1-AS1 may function as a crucial target lncRNA for the AML treatment.

In the present work, we screened transcriptome profiles of differentially expressed lncRNAs in AML and observed that the expression of SATB1-AS1 was most significantly enhanced versus other lncRNAs in PBMC of AML patients. Furthermore, we substantiated SATB1-AS1 elevated Adr and Cyt resistance in AML cells. SATB1 was found to be dysregulated commonly (22 out of 23 patients) between primary and relapsed AML samples [29]. However, there was no related report on whether SATB1-AS1 affects chemotherapy resistance in AML. We presented data confirming that SATB1-AS1 expression in AML patients was enhanced relative to that observed in normal controls, and that SATB1-AS1 has the potency to reduce the chemosensitivity of AML both *in vitro* and *in vivo*.

LncRNAs have been implicated to function as ceRNAs to disrupt miRNA activity through sequestration, contributing to enhanced expression of the target gene of the miRNA [29]. In this study, we screened out OAS2 to be significantly enhanced in AML samples and linked to overexpression of SATB1-AS1. Later, we predicted that SATB1-AS1 was localized in the cytoplasm through an online website and corroborated that it may function as a miRNA sponge using FISH. The online software projected that miR-580 both interacted with SATB1-AS1 and OAS2, which was further verified using luciferase reporter assays. Overexpression of miR-580 inhibited cell motility in breast cancer by downregulating TWIST1, implying that miR-580 functioned as a tumor suppressor [30]. Moreover, circRAB3IP has been revealed to act as the sponge of miR-580-3p to promote TWIST1 expression in osteosarcoma [31]. However, the correlation between miR-580 and AML has not been explicated. Our data illustrated that SATB1-AS1 upregulated chemotherapy resistance of AML cells to Adr and Cyt by inhibiting miR-580 and restoring OAS2. To substantiate this hypothesis, we conducted rescue experiments. Either miR-580 downregulation or OAS2 upregulation abrogated the inhibitory role of SATB1-AS1 knockdown on AML cell chemoresistance. Particularly, OAS has been linked to immune-regulatory functions that facilitate autoimmune disorders, infectious diseases as well as cancers [32]. For example, miR-340 weakened cellular antiviral immunity by binding to and repressing the expression of OAS2 [33]. Moreover, OAS2 was monitored to be highly enriched and overexpressed in the hepatitis C virus-infected hepatocellular carcinoma [34]. The association between OAS2 and chemoresistance has been rarely investigated, which highlights the novelty of our study.

Further exploration identified that the GSK3β/β-catenin signaling was engaged in the modulation of chemoresistance by the SATB1-AS1/miR-580/OAS2 axis, as evidenced by diminished GSK3β and enhanced β-catenin expression in drug-resistant cells and the opposite trends after the delivery of si-SATB1-AS1. Similarly, miR-302a potentiated the chemosensitivity of AML cells to etoposide at least partially *via* the GSK3β/β-catenin axis [35]. In addition, rosmarinic acid enhanced cisplatin sensitivity of melanoma cells through mediating the ADAM17/EGFR/AKT/GSK3β axis [36]. More relevantly, pleiotrophin elevated chemoresistance to Adr in osteosarcoma cells by inducing the GSK3β/β-catenin signaling [37]. More specifically, the GSK-3β inhibitor AR-A014418 held the capacity of eliminating the repressive role of si-SATB1-AS1 on AML cell resistance in the current report. Consistent findings were reported before where inactivation of GSK3β partially reversed the TRIM24 knockdown-mediated antitumor effects in AML cells [38].

## Conclusion

Taken together, a significant surge of SATB1-AS1 expression was observed in PBMC derived from AML patients and AML cells. SATB1-AS1 knockdown enhanced the sensitivity to Adr and Cyt, inhibited viability, but induced apoptosis of drug-resistant AML cells. OAS2, a target of miR-580, could be promoted by SATB1-AS1. In a word, SATB1-AS1 contributed to Adr and Cyt resistance and progression of AML cells by upregulating OAS2 via mediating miR-580. Our study provided a possible strategy to antagonize Adr and Cyt resistance and malignancy in AML.

## Supplementary Material

Supplemental Material

## Data Availability

The datasets used and/or analyzed during the current study are available from the corresponding author on reasonable request.
